# Editorial: Microbial ecological and biogeochemical processes in the soil-vadose zone-groundwater habitats, volume III

**DOI:** 10.3389/fmicb.2025.1763007

**Published:** 2026-01-12

**Authors:** Huai Li, Zifang Chi, Jiuling Li, Yihao Luo

**Affiliations:** 1 Northeast Institute of Geography and Agroecology, Chinese Academy of Sciences, Changchun, China; 2 Key Lab of Groundwater Resources and Environment, Ministry of Education, Jilin University, Changchun, China; 3 School of Civil and Environmental Engineering, Queensland University of Technology, Gardens Point, Brisbane, QLD, Australia; 4 Engineering Lab for Water Pollution Control and Resources Recovery of Jilin Province, School of Environment, Northeast Normal University, Changchun, China

**Keywords:** biogeochemical processes, groundwater, habitats, soil, vadose zone

Microorganisms are pivotal in regulating biogeochemical cycles and performing critical functions in soil, vadose zone, and groundwater habitats (Chi et al., 2022; Song et al., 2024). Microbial communities in these habitats respond and shift rapidly to adapt to environmental changes (Li et al., 2024; Liang et al., 2023). Both biotic and abiotic conditions shape microbial composition and activity, which in turn affect key ecosystem processes (Li et al., 2022; Chi et al., 2025). Environments such as natural wetlands, landfills, composts, vadose zones, and saturated aquifers are all parts of the broader soil-vadose zone-groundwater. The aim of this volume is to collect innovative research that advances our understanding of microbial ecological processes in these habitats and explore the possibilities of developing sustainable environmental management. Nine articles included in this Research Topic have undergone rigorous peer review and have been selected for their contributions to these areas of study.

Wang X. et al. revealed the pronounced functional disparities among *Artemisia scoparia, Aeluropus sinensis* and *Suaeda salsa* communities. The herbaceous plant community significantly affected the physicochemical properties of the 0–20 cm soil layer. *Suaeda salsa, Artemisia scoparia*, and *Phragmites australis* communities were the most effective communities for comprehensive soil quality improvement on Yellow River Island.

Chi, Zhang et al. demonstrated that the ultra-high-temperature (UHT) composting bioaugmented with a high-dose microbial inoculum (CT2) could achieve highly efficient co-remediation of heavy metals and antibiotics in livestock manure. This process significantly promoted the passivation of Cu and Zn and achieved near-complete degradation (>99.4%) of tetracycline (TC), oxytetracycline (OTC), and chlortetracycline (CTC).

Chi, Li et al. found that the addition of small particle activated carbon (S-AC, 2–4 mm) could increase the pH value and organic matter of organic fertilizers, and reduce the cation exchange capacity (CEC) value, but had no significant effect on the electrical conductivity (EC) value. S-AC had high mechanical strength (compressive strength 4.014 MPa), low loss rate, and high recovery rate, showing the best remediation performance. S-AC performed the best in the removal of heavy metals (total and available Cu and Zn) and the degradation of antibiotics [tetracycline (TC) and ciprofloxacin (CIP)].

Bagwell et al. analyzed the water samples (background and down-gradient groundwater) from 14 coal combustion management sites across 4 physiographic regions in the United States for water chemistry and microbiology. The chemistry distinguished sample types by standard water quality metrics, alkali earth metals, selenium, boron, and fluoride. The single coal combustion products (CCP) management site was confirmed to have groundwater impacted by CCP leachate. At this site, 9 genera (>0.5% relative abundance) were exclusive to CCP porewater and downgradient groundwater. The potential for N and S biogeochemical transformations and 1-C metabolism was consistent with a reducing environment, as evidenced by low ORP and depleted ${\text{SO}}_{4}^{2-}$.

Liu et al. found that the thinning promoted the growth of *Ulmus pumila* but did not significantly promote the growth of *Salix matsudana* or *Fraxinus chinensis*, which provided a scientific basis for optimizing forestry management strategies. In addition, the thinning played an important role in soil improvement and soil and water conservation by improving soil physical properties, especially in areas with severe soil erosion. Understanding the effect of thinning on the soil nutrient content was helpful for ecological restoration and biodiversity improvement.

Koster et al. discovered that the soil bacterial alpha functional diversity were positively correlated with patch area and number of vascular plant species in the forest floor vegetation layer. However, soil bacterial beta functional diversity was primarily driven by patch area and soil physicochemical properties. The positive effect of patch area (biogeographic effect) on soil bacterial functional diversity may be primarily through stabilization of environmental conditions, as the amplitude of environmental fluctuations is reduced on larger plots compared to smaller ones.

Chen et al. investigated the influence of uranium contamination on microbial community composition in mine tailing environments. Mining operations and remediation efforts resulted in geochemical heterogeneity across different sites. Despite these varied conditions, microbial communities remained adaptable. The uranium concentration was the primary driver of microbial community structure. The simpler, less interconnected microbial networks were present under high uranium conditions, while intermediate and relatively low uranium levels supported more complex assemblages. The stochastic processes such as dispersal limitation dominated community assembly in high-uranium environments, whereas deterministic processes, including both heterogeneous and homogeneous selection, were more significant in those environments with moderate to low uranium concentrations. The authors conclude that at lower uranium concentrations cooperation among microbial taxa may prevail, while at higher concentrations, microbes may shift to a competitive “fight for their own” strategy.

Wang Z. et al. unveiled that the compositions and diversities of abundant and rare subcommunities were notably altered in crab-bioturbated sediments. The crab bioturbation substantially modified the interaction patterns of rare bacteria, whereas its influence on abundant bacteria was comparatively minor. The assembly processes of abundant subcommunities were primarily influenced by stochastic processes, while rare subcommunity assemblies were collectively shaped by both stochastic and deterministic processes.

Hao et al. revealed that both stabilization and freeze–thaw treatment could reduce the leaching concentration of Pb and the proportion of available Pb, increase the bioavailability of Pb in the gastric phase (simulated gastric juice), but decrease in the intestinal phase (intestinal fluid). The dominant bacterial phylum in the soil changed to Firmicutes, the dominant bacterial genus changed to Bacillus. The bioavailability of Pb was related to soil pH, cation exchange capacity (CEC), soil organic matter (SOM), soil moisture content (SMC), Pb (leaching, acid soluble state, oxidation state, residual state), and types of microorganisms in soil.

We think that all accepted articles in this Research Topic will provide new knowledge on microbial processes in soil-vadose zone-groundwater habitats.
